# Sudden cardiac arrest in infants and children: proposal for a diagnostic workup to identify the etiology. An 18-year multicenter evaluation in the Netherlands

**DOI:** 10.1007/s00431-023-05301-9

**Published:** 2023-10-27

**Authors:** Ashley M. Bakker, Marijn Albrecht, Bas J. Verkaik, Rogier C. J. de Jonge, Corinne M. P. Buysse, Nico A. Blom, Lukas A. J. Rammeloo, Judith M. A. Verhagen, Maaike A. Riedijk, Sing C. Yap, Hanno L. Tan, Janneke A. E. Kammeraad

**Affiliations:** 1 Department of Pediatric Cardiology, Erasmus MC Sophia Children’s Hospital, Postbus 2060, 3000 CB Rotterdam, The Netherlands; 2 Department of Neonatal and Pediatric Intensive Care, Division of Pediatric Intensive Care, Erasmus MC Sophia Children’s Hospital, Rotterdam, The Netherlands; 3 Heart Center, Department of Clinical and Experimental Cardiology, Amsterdam UMC, University of Amsterdam, Cardiovascular Sciences, Amsterdam, The Netherlands; 4 The Center for Congenital Heart Disease Amsterdam–Leiden, Amsterdam, The Netherlands; 5 Department of Pediatric Cardiology, Amsterdam University Medical Center, Location AMC, Amsterdam, The Netherlands; 6 Department of Clinical Genetics, Erasmus MC, University Medical Center Rotterdam, Rotterdam, The Netherlands; 7 Department of Pediatric Intensive Care, Emma Children’s Hospital, Amsterdam University Medical Center, Amsterdam, The Netherlands; 8 Department of Cardiology, Erasmus MC, Rotterdam, The Netherlands; 9 Netherlands Heart Institute, Utrecht, The Netherlands

**Keywords:** Sudden cardiac arrest, Pediatrics, Etiologies, Diagnostic workup, Cardiac history

## Abstract

Sudden cardiac arrest (SCA) studies are often population-based, limited to sudden cardiac death, and excluding infants. To guide prevention opportunities, it is essential to be informed of pediatric SCA etiologies. Unfortunately, etiologies frequently remain unresolved. The objectives of this study were to determine paediatric SCA etiology, and to evaluate the extent of post-SCA investigations and to assess the performance of previous cardiac evaluation in detecting conditions predisposing to SCA. In a retrospective cohort (2002–2019), all children 0–18 years with out-of-hospital cardiac arrest (OHCA) referred to Erasmus MC Sophia Children's Hospital or the Amsterdam UMC (tertiary-care university hospitals), with cardiac or unresolved etiologies were eligible for inclusion. SCA etiologies, cardiac and family history and etiologic investigations in unresolved cases were assessed. The etiology of arrest could be determined in 52% of 172 cases. Predominant etiologies in children ≥ 1 year (n = 99) were primary arrhythmogenic disorders (34%), cardiomyopathies (22%) and unresolved (32%). Events in children < 1 year (n = 73) were largely unresolved (70%) or caused by cardiomyopathy (8%), congenital heart anomaly (8%) or myocarditis (7%). Of 83 children with unresolved etiology a family history was performed in 51%, an autopsy in 51% and genetic testing in 15%. Pre-existing cardiac conditions presumably causative for SCA were diagnosed in 9%, and remained unrecognized despite prior evaluation in 13%.

*Conclusion*: SCA etiology remained unresolved in 83 of 172 cases (48%) and essential diagnostic investigations were often not performed. Over one-fifth of SCA patients underwent prior cardiac evaluation, which did not lead to recognition of a cardiac condition predisposing to SCA in all of them. The diagnostic post-SCA approach should be improved and the proposed standardized pediatric post-SCA diagnostics protocol may ensure a consistent and systematic evaluation process increasing the diagnostic yield.

**Table:** 

**What is Known:** *• Arrests in infants remain unresolved in most cases. In children > 1 year, predominant etiologies are primary arrhythmia disorders, cardiomyopathy and myocarditis.* *• Studies investigating sudden cardiac arrest are often limited to sudden cardiac death (SCD) in 1 to 40 year old persons, excluding infants and successfully resuscitated children.*	
**What is New:** *• In patients with unresolved SCA events, the diagnostic work up was often incompletely performed.* *• Over one fifth of victims had prior cardiac evaluation before the arrest, with either a diagnosed cardiac condition (9%) or an unrecognized cardiac condition (13%).*

**Graphical Abstract:**

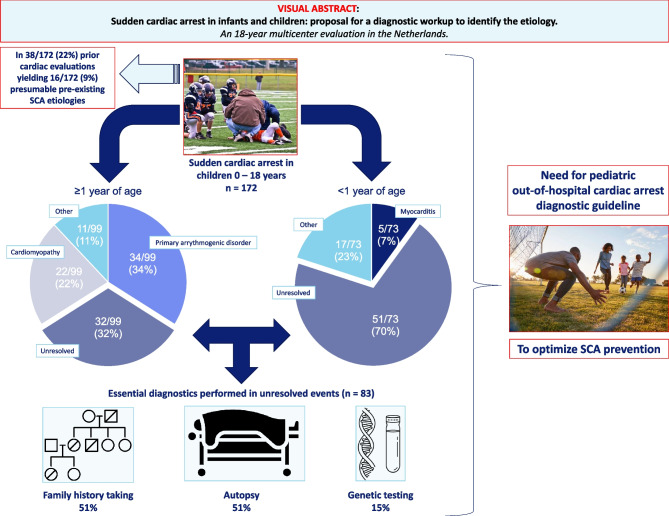

**Supplementary Information:**

The online version contains supplementary material available at 10.1007/s00431-023-05301-9.

## Introduction

Sudden cardiac arrest (SCA) in children is estimated to account for 10% of the total mortality in the Dutch pediatric population [1]. In case of survival, the child and caregivers may face devastating consequences, varying from mild to severe, both in the short- and long-term [2].

Reported SCA incidences in children and young adult range from 0.5 to 3.9 per 100,000 person-years [1, 3–9]. Studies investigating SCA are often limited to sudden cardiac death (SCD) in 1 to 40 year old persons, excluding infants and successfully resuscitated children [4, 6, 9]. Still, within the pediatric population, infants have the highest out-of-hospital cardiac arrest (OHCA) incidence and lowest survival rates [3]. SCA in infants are often presumed to be multifactorial in etiology in analogy to Sudden Infant Death Syndrome/Sudden Unexplained Death in Infancy/Apparent life-threatening event (SIDS/SUDI/ALTE). However, a small percentage of these cases is likely a result of an inherited cardiac disease and their identification and early detection in relatives may have lifesaving consequences [10–14]. In children > 1 year, predominant etiologies are primary arrhythmia disorders, cardiomyopathy and myocarditis [4, 5, 7, 15, 16].

Postmortem genetic testing may aid in etiology identification [4, 16–19]. Identifying a (likely) pathogenic gene variant allows cascade screening within families, leading to diagnoses in pre-symptomatic carriers and potential prophylactic treatment and lifestyle modifications that may prevent new events [17, 20, 21]. Yet, after extensive postmortem investigations, 30–40% of events remain unresolved, with the highest unresolved rates found in children and young adults (1–5 and 16–20 years), warranting further investigation in these age groups [4, 6].

It is crucial to gain insight into etiologies to guide opportunities for preventing SCA. The present multicenter study investigates etiologies of pediatric SCA and assesses the diagnostic investigations performed in the SCA subgroup with unresolved etiology. In addition, we investigate the rate and diagnostic yield of cardiac evaluation performed before pediatric SCA.

## Methods

### Study design and setting

This retrospective observational multicenter study was conducted at two tertiary-care university hospitals in the Netherlands: the Erasmus MC Sophia Children’s Hospital (Erasmus MC) and the Amsterdam University Medical Center (AUMC). The catchment area of these hospitals covers 40% of the Dutch population. Ethical approval was waived by the local Ethics Committees of the Erasmus MC and AUMC in view of the retrospective nature of the study and all the procedures being performed were part of the routine care (MEC-2021-0618 and MEC-2017-260) and the research was conducted in accordance with the 1964 Helsinki Declaration and its later amendments.

### Inclusion criteria and definitions

Definitions are specified in Supplementary Table 1 [18]. The study population consisted of children 0–18 years of age with treated OHCA referred to one of the participating university hospitals between January 2002 and August 2019. Only OHCA with a proven cardiac or unresolved etiology were included. Submersion injury in children > 8 years old was also included when these children were presumed to be able to swim. Perinatal death (within 24 h after delivery) and non-cardiac etiologies such as trauma, cerebral bleeding, sepsis and respiratory failure were excluded. In case of uncertainty about presumed non-cardiac etiology, consensus was reached by a team of specialized (pediatric) cardiologists (J.A.E.K., L.A.J.R.) and a pediatric intensivist (C.M.P.B.).

### Data collection

Children were identified from two prospective population-based resuscitation databases. 1) The Erasmus MC database includes all resuscitated pediatric OHCA patients from 2002 onwards in the province of South Holland [22]. 2) The AmsteRdam REsuscitation STudies (ARREST) database, including all OHCA patients from 2005 and onwards in the province of North Holland [1]. Collected data comprised: (1) basic child characteristics, e.g. age, gender, (2) SCA characteristics, e.g. initial cardiac rhythm, etiology and return of circulation (ROC), (3) medical history, e.g. (cardiac) diagnoses, cardiac evaluation, (cardiac) surgeries before the SCA, (4) family history of cardiac disease or epilepsy, (5) post-SCA diagnostic evaluation, e.g. autopsy report, toxicology, blood culture and genetic analysis. Genetic analysis was determined for the proband, but not for family members due to the absence of informed consent for access to their medical records.

Based on this information, the etiology of SCA was determined for each child and categorized as primary arrhythmogenic disorder, cardiomyopathy, congenital heart anomaly, and other cardiac causes (cardiac neoplasms, Kawasaki disease and myocarditis) or unresolved etiology. Idiopathic ventricular fibrillation (IVF) was diagnosed with documented VF and exclusion of known cardiac, respiratory, metabolic, and toxicological etiologies through clinical evaluation [23]. Positive family history for a possible inherited cardiac condition (ICC) was defined as one or more first or second-degree relative(s) with either i. unexplained death or SCA < 40 years of age, ii. primary arrhythmia disorders, or iii. non-ischemic cardiomyopathy. Primary arrhythmia disorders included inherited syndromes or implantable cardioverter-defibrillator (ICD) or pacemaker implantation (< 40 years of age).

### Statistical analysis

Categorical variables are reported as frequencies (n) and percentages (%). Continuous variables are reported as medians with first and third quartiles (Q1;Q3) or means with standard deviations (SD) depending on the normality of distribution. Baseline characteristics and outcomes are evaluated by age group (< 1 year and ≥ 1 year). Depending on applicability, differences between age groups were assessed with a Fisher exact test and an unpaired t-test or Mann–Whitney U test. Statistical significance was considered with a two-tailed p-value < 0.05. All analyses were conducted using SPSS software (IBM Corp. Released 2021. IBM SPSS Statistics for Windows, Version 28.0. Armonk, New York).

## Results

### Basic characteristics

**Fig. 1:**
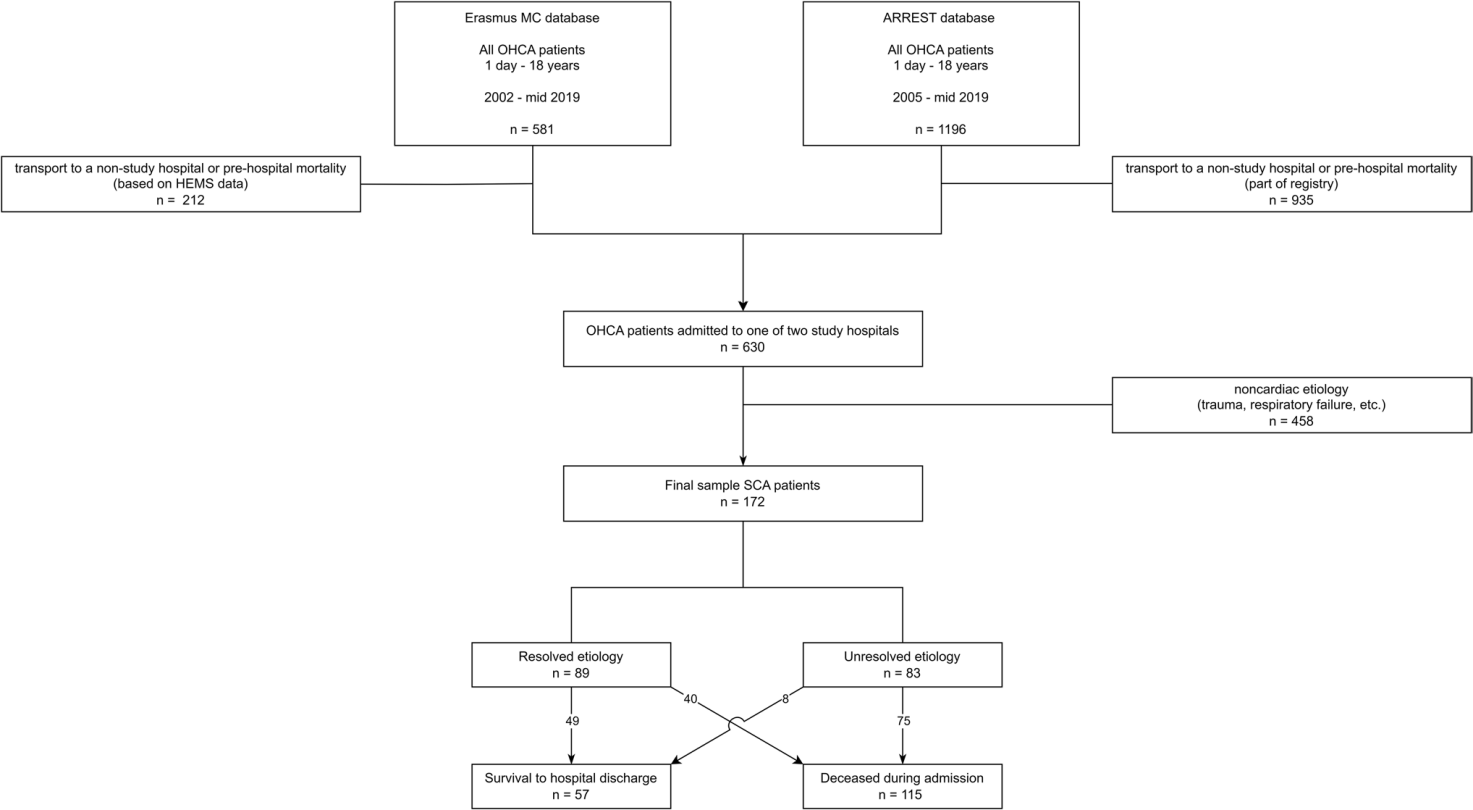
Overview of inclusion. Abbreviations: OHCA = Out-of-hospital cardiac arrest, ARREST AmsteRdam. REsuscitation Studies, HEMS = Helicopter Emergency Medical Services

An inclusion overview is provided in Fig. 1. The pediatric OHCA population consisted of 1777 children, of whom 1147 died on scene or were transferred to a non-study hospital. Of the children admitted to one of the two study hospitals, 458 were excluded because of a non-cardiac etiology resulting in a final sample of 172 children. The baseline characteristics are presented in Table 1. The median age of the study population was 2.3 years (Q1;Q3 0.3–12.2), and 64% were male. The majority of children (109/172, 63%) presented with a non-shockable rhythm, especially in younger children (65/73, 89% < 1 year versus 44/99, 44% ≥ 1 year; p < 0.001). Overall, 57/172 children (33%) survived to hospital discharge.

**Table 1:** Baseline characteristics stratified by age

	Overall			Age < 1 year			Age ≥ 1 year			
	(n = 172)			(n = 73)			(n = 99)			
	n^a^			n^a^			n^a^			p-value^d^
**Child characteristics**										
Age (years)^b^	172	2.3	0.3–12.2	73	0.3	0.1–0.4	99	11.3	5.2–14.9	< 0.001
Male gender^c^	172	110	64%	73	47	64%	99	63	64%	> 0.999
**Event characteristics**										
Initial rhythm^c^	172			73			99			
*Shockable (VF/VT)*	172	60	35%	73	6	8%	99	54	55%	< 0.001
*Unknown/ROSC before EMS arrival*	172	3	2%	73	2	3%	99	1	1%	0.575
*Non-shockable*	172	109	63%	73	65	89%	99	44	44%	< 0.001
- *Asystole*	109	66		65	37		44	29		0.007
- *PEA*	109	3		65	0		44	3		0.263
- *Bradycardia*	109	17		65	13		44	4		0.004
- *Other*	109	3		65	1		44	2		> 0.999
- *Unknown non-shockable (AED) rhythm*	109	20		65	14		44	6		0.014
**Outcome**										
Sustained ROC^c^	172	101	59%	73	38	52%	99	63	64%	0.159
Survival to hospital discharge^c^	172	57	33%	73	10	14%	99	47	47%	< 0.001

*SCA* Sudden cardiac arrest, *VF* Ventricular fibrillation, *VT* ventricular tachycardia, *RO(S)C* Return of (spontaneous) circulation, *EMS* Emergency medical services, *AED* Automated external defibrillator, *PEA* Pulseless electrical activity

^a^Number of subjects in whom the variable was obtained

^b^Median (interquartile range)

^c^Number of subjects (%)

^d^p-Value (age < 1 year vs. age ≥ 1 year): Fisher's exact test for dichotomous data, Mann–Whitney U test for continuous data

### Etiologies

The etiologies of all 172 events are presented in Table 2. Of 73 infant events, 22 were resolved (30%). Predominant etiologies were cardiomyopathies (6/73, 8%), congenital heart anomalies (6/73, 8%) and other cardiac etiologies (7/73, 10%), in particular myocarditis. Of 99 events in children ≥ 1 year old, 67 were resolved (68%). Predominant etiologies were primary arrhythmogenic disorders (34/99, 34%) and cardiomyopathies (22/99, 22%). In 48% of the total population, the etiology of the SCA event remained unresolved (51/73, 70% in the < 1 year age group versus 32/99, 32% in the ≥ 1 year age group; p < 0.001). Furthermore, among non-survivors, 65% of events (75/115) remained unresolved, while this proportion was 14% (8/57) among survivors.

**Table 2:** Etiology of SCA by age

	Overall			Age < 1 year			Age ≥ 1 year			
	(n = 172)			(n = 73)			(n = 99)			
	n^a^			n^a^			n^a^			p-value^c^
**Primary arrhythmogenic disorders**^**b**^	172	37	22%	73	3	4%	99	34	34%	< 0.001
Idiopathic VF^b^	37	11		3	1		34	10		0.026
CPVT^b^	37	10		3	0		34	10		0.005
LQTS^b^	37	5		3	0		34	5		0.073
WPW-syndrome^b^	37	5		3	0		34	5		0.072
Other^b^	37	6		3	2		34	4		> 0.999
**Cardiomyopathy**^**b**^	172	28	16%	73	6	8%	99	22	22%	0.020
Hypertrophic CMP^b^	28	18		6	3		22	15		0.022
Dilated CMP^b^	28	7		6	3		22	4		> 0.999
Other^b^	28	3		6	0		22	3		0.262
**Congenital heart anomaly**^**b**^	172	9	5%	73	6	8%	99	3	3%	0.171
**Other cardiac causes**^**b**^	172	15	9%	73	7	10%	99	8	8%	0.466
Myocarditis^b^	15	11		7	5		8	6		> 0.999
Other^b^	15	4		7	2		8	2		> 0.999
**Unresolved**^**b**^	172	83	48%	73	51	70%	99	32	32%	< 0.001

*VF* Ventricular fibrillation, *CPVT* Catecholaminergic polymorphic ventricular tachycardia, *LQTS* Long QT syndrome, *WPW-syndrome* Wolff-Parkinson-White syndrome, *CMP* Cardiomyopathy

^a^Number of subjects in whom the variable was obtained

^b^Number of subjects (%)

^c^p-Value (age < 1 year vs. age ≥ 1 year): Fisher's exact test for dichotomous data

### Diagnostic evaluation of unresolved events

Post-SCA diagnostics appeared to be often incompletely performed in 83 unresolved events as presented in Table 3. A family history was documented in 51% (42/83). In 75 deceased children, autopsy was offered to all caregivers, but accepted in 51% (42/83). Lastly, in 15% (10/68), cardiogenetic testing was performed.

**Table 3:** Post-SCA diagnostic evaluation of unresolved SCA events

	Unresolved events (n = 83 in total, n = 75 deceased during admission)			
	n^a^	n^b^		Missing
**Toxicology screening**^**c**^	74	37	50%	9
**Blood culture testing**^**c**^	76	55	72%	7
**Family history documented**^**c**^	83	42	51%	0
**Postmortem total body MRI**^**c**^	64	13	20%	11
**Autopsy**^**c**^	70	36	51%	5
**Genetic testing of proband**^**c**^	68	10	15%	15

*MRI* Magnetic resonance imaging

^a^Number of subjects in whom the variable was obtained

^b^Number of subjects in whom the investigation was performed

^c^Number of subjects (%)

### Medical and family history in relation to SCA etiology

**Fig. 2:**
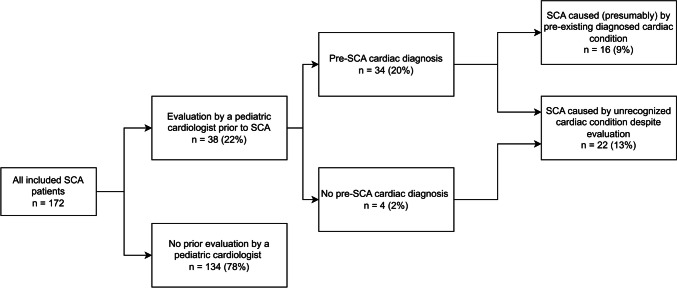
Prior cardiac evaluation in relation to etiology of SCA. Abbreviations: SCA = Sudden Cardiac Arrest

An overview of the children that underwent cardiac evaluation prior to SCA is presented in Fig. 2. Evaluation by a pediatric cardiologist before the SCA event took place in 38/172 (22%) children. From these, 34/38 (89%) children were diagnosed with a cardiac condition, which was not always a condition predisposing to SCA. A cardiac condition predisposing to SCA was diagnosed in 16/38 (42%) children, while in 22 children (58%), the cardiac condition that presumably caused SCA had remained undiagnosed despite prior cardiac evaluation.

A more detailed summary of medical and family history is presented in Supplementary Table 2. Notably, 5/37 (14%) and 9/28 (28%) of children in the primary arrhythmogenic disorder and cardiomyopathy groups respectively had been diagnosed with the presumable SCA etiology. In addition, seven children (7/37, 19%) with SCA due to a primary arrhythmogenic disorder, suffered a prior syncope that did not result into a referral for evaluation by a pediatric cardiologist. In the unresolved etiology cases, 13% (11/83) had prior cardiac evaluation, mostly identifying unrelated congenital cardiac anomalies.

Among all SCA cases, twenty-five children (25/170, 15%) had a neurologic condition prior to SCA, half of whom had epilepsy (12/25, 48%). A positive family history of ICC was present in 13% (15/118).

## Discussion

In this study of pediatric patients with out-of-hospital cardiac arrest, the etiology of arrest could be established in 52% of cases (89/172). Arrest at age < 1 year was associated with a lower proportion of the cause of arrest being identified (30%, 22/73). In patients with unresolved SCA events, the diagnostic work up was often incompletely performed. Twenty-two percent of victims (38/172) had undergone cardiac evaluation before the arrest, with either a diagnosed cardiac condition (9%, 16/172) or an unrecognized cardiac condition (13%, 22/172).

### Etiologies

Comparison of etiologies to the existing literature is difficult due to the scarcity of literature on infant SCA and differences in inclusion criteria (predominantly SCD and hospital admission versus population-based). In the present study, predominant etiologies in children aged ≥ 1 year were inherited cardiac diseases, including primary arrhythmia disorders (34%, 34/99) and hypertrophic cardiomyopathy (15%, 15/99), and 32% (32/99) unresolved cases, which is comparable to previous literature [4, 5, 7, 15, 16, 24].

However, the etiology remained unresolved for infant cases in 70% (51/73), as was recognized in the only prior SCD cohort including infants which described 90% of SUDI [25]. Of the remaining resolved infant cases, 40% (9/22) had a potential inherited cardiac condition, supporting previous studies reporting causative cardiogenetic factors in up to 10% of SUDI cases [10–12, 26]. In the existing literature on pediatric OHCA, infants form the largest population and have the highest unresolved SCD rate underlining the importance of their inclusion in pediatric SCA studies [3, 4]. Infant arrests often occur unwitnessed during sleep at home, potentially causing a malignant ventricular arrhythmia to devolve into an asystole at the time of arrival of emergency medical services [3]. Compared to a shockable rhythm, a non-shockable presenting rhythm is associated with lower survival chances [22]. Most infants in our study presented with a non-shockable rhythm, and 86% (60/73) died. The unavailability of an electrocardiographic phenotype impairs diagnosing a potential (inherited) cardiac arrhythmia and contributes to the high proportion of unresolved etiologies. Furthermore, the finding that 7% (5/73) of the infant population suffered SCA due to myocarditis has not been previously described. This might be an underestimation because the diagnosis of myocarditis, especially postmortem, is complicated: only in 41% (21/51) an autopsy was performed and if morphologic myocardial inflammation was present, a micro-organism that may cause the disease, can often not be demonstrated.

Unlike most previous studies, we included (33%, 57/172, surviving) hospital-admitted SCA cases. This allowed for extensive diagnostic (cardiac) phenotyping, thereby increasing the chance of diagnosing the condition that caused SCA compared to the SCD population-based approach. The proportion of resolved etiologies in the surviving versus deceased patients was 55% (32/57) and 10% (12/115), respectively. The presenting rhythm was of utmost importance, with a 65% (39/60) diagnostic yield in presenting shockable rhythm versus 2% (2/109) in a non-shockable rhythm.

Alapati et al. described the only other pediatric hospital-admitted SCA cohort consisting of 44 children without medical history [29]. They described a survival rate of 50% (21% of infants and 72% ≥ 1 year) and 90% unresolved infant cases versus 16% unresolved ≥ 1-year cases [24]. The survival rate and the rate of resolved etiologies ≥ 1 year are higher than in the present study. This is an unexpected difference since Alapati et al. excluded patients with known medical conditions, such as cardiac diseases, who were included in the present study. This difference may be partly explained of the inclusion of “secondary LQTS” and ruptured arteriovenous malformation in the brain, who would have been excluded from our study [24].

### Post-SCA diagnostics

Recent guidelines have focused on investigating patients with sudden unexplained death and their families [18]. One of our study's critical and awareness-raising findings is that essential diagnostics were often not performed in unresolved cases. In natural death etiologies, the choice for an autopsy is up to the child's caregivers, and a total body MRI may be offered when they do not give permission for autopsy. As many emotional and cultural considerations influence this decision and are not likely to change much over a short time frame, a significant increase in the autopsy rate is not expected. However, increasing the rate of blood culturing, toxicology screening, and obtaining a family history and cardiogenetic testing must be systematically considered. For example, the crucial step of taking a thorough familial history should be performed in every patient to streamline additional investigations into potential cardiac predispositions, in particular genetic testing.

**Fig. 3:**
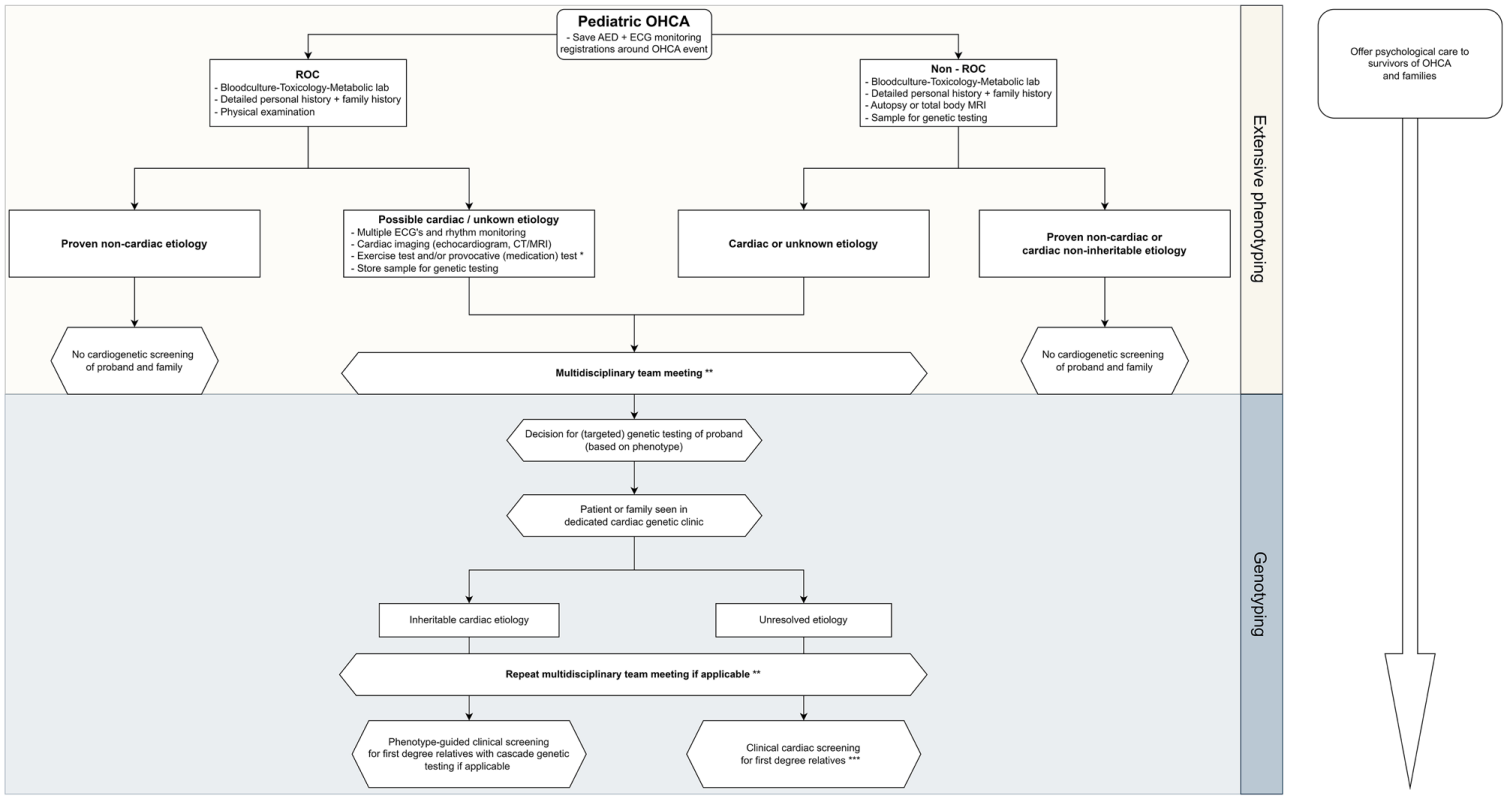
Proposed post-SCA diagnostic protocol in pediatric SCA. Adapted from Stiles et al. and Erasmus MC protocol [18]. Abbreviations: OHCA = Out-of-hospital cardiac arrest, AED = Automated external defibrillator, ECG = Electrocardiogram, ROC = Return of Circulation, MRI = Magnetic Resonance Imaging, CT Computed Tomography. * In brain death or withdrawal of life sustaining therapy cases, consider provocative medication testing eg ajmaline and epinephrine, to unmask specific ECG phenotypes. ** Multidisciplinary team represents: pediatric and adult cardiologist, clinical and molecular geneticist, cardiac imaging, pathology and others if required. *** Negative DNA diagnostics do not rule out an ICC. Keep patient and 1st degree family members under periodic re-evaluation

Promising in that regard is postmortem genetic testing [16]. Bagnall et al. showed in their three-year prospective population-based study that, in unexplained SCD cases, autopsy investigation combined with genetic testing revealed a likely etiology in 27% of cases [4]. A definite clinical diagnosis was established in 13% of followed-up first-degree relatives [4]. Evaluating the yield of clinical and genetic testing in relatives was not an objective of our study. Still, others have shown diagnosis of ICC in up to 40% of families with ≥ 1 sudden and unexplained death victim [20, 27]. The importance of these findings for relatives in preventing new SCA cases needs no explanation. However, others also recently described difficulty performing structured investigations after SCA, reporting the collection of blood samples for genetic analyses in only 1% of SCA victims between 18–45 years [28]. In our centers, these findings have resulted in the development and implementation of a standardized pediatric post-SCA diagnostics protocol (Fig. 3, adapted from Stiles et al. [18], and Supplementary Table 3). A structured evaluation of children with SCA may increase the proportion of non-cardiac etiologies in OHCA. In the Erasmus MC a trend was observed towards decreasing rates of unresolved cases when the period before and after the introduction of the protocol are compared; 58% (55/95) prior and 17% (3/18) after (p = 0.001). In addition, it may increase the contribution of ICC, providing the opportunity for cascade screening and, in the case of carriership, preventive care for relatives, potentially preventing new SCA cases.

### Prior evaluation or diagnosis

The finding that a substantial proportion of children suffered SCA presumably caused by a pre-SCA diagnosed cardiac condition or had undergone a prior cardiac evaluation without recognizing a condition with increased risk for SCA has not been described before in a pediatric population. This study was not designed to conclude whether these events would have been preventable nor whether all necessary investigations to exclude an underlying unrecognized cardiac etiology had been performed. However, the results suggest that not in all evaluated children a SCA risk can be recognized and if a SCA risk was recognized, not all arrests can be prevented. Treatment of children with a cardiac condition predisposing to SCA is challenging and needs a deliberate assessment of SCA risk against the potential drawbacks of mitigating lifestyle changes and treatment [29–31]. Moreover, the risk of SCA is multifactorial and dependent on the specific disease, variable penetration and expression, available treatment options and therapy adherence.

However, most children did not have a pre-existing diagnosed cardiac condition, which aligns with previous reports [32, 33]. Only few patients had symptoms like syncope and epilepsy was diagnosed in 7% (12/170) prior to the event. Epilepsy is difficult to interpret in relation to SCA; on one hand are children with epilepsy at increased risk of sudden death especially in case of refractory epilepsy [34, 35]. This might be the etiology of SCA in a proportion of the unresolved cases. On the other hand may primary arrhythmogenic disorders present as actual seizures that can be misdiagnosed as epilepsy, and also overlap syndromes between epilepsy and LQTS exist [35–37].

Recognition of SCA risk in asymptomatic children might be challenging for several reasons. First, the development of certain cardiac conditions with increased SCA risk is not predictable (e.g. myocarditis). Recognition will depend on the presence and recognition of symptoms of which SCA can be the first. Second, other cardiac conditions with more chronic appearance (e.g. cardiomyopathy and primary arrhythmia syndromes) often develop a phenotype somewhere between childhood and adolescence. Recognition therefore requires repeated cardiac evaluations during childhood. Third, specific provocative investigations are required to unmask certain cardiac conditions. These investigations (e.g. exercise testing and ECG during fever) are not routinely performed during a general cardiac work up which may lead to false reassurance [38, 39]. This might partly explain our population's 19% proportion of unrecognized primary arrhythmogenic disorders despite cardiac evaluation.

Improvement might be expected through a standardized cardiac evaluation in suspicious children, e.g. with a suspected family history or complaints of non-vasovagal syncope or atypical epilepsy, including exercise testing and potentially other provocative testing [30, 33–35, 40]. Increased societal awareness of suspected family history of ICC and symptoms that might indicate a potential increased risk for SCA, might help to identify the children for whom this evaluation is appropriate. Alapati et al. described 32% of SCA patients to be potentially identified as at risk for SCA by a questionnaire combined with ECG [24].

In the past decade, a significant yield in determining a population at risk for SCA has been achieved by cascade screening in families carrying a (likely) pathogenic DNA variant. However, in clinical practice, some parents still do not want their children to undergo DNA diagnostics for a familial variant due to various considerations ranging from appreciating their child’s autonomy to concerns about problems obtaining their mortgage in future. Future investigations must focus on improving the yield of recognizing children at risk for SCA by medical professionals and creating societal awareness.

### Strengths and limitations

Our study aimed to describe a population of pediatric SCA patients, including infants, in relation to prior evaluation by a cardiologist and pre-existing cardiac medical history. The catchment area of this multicenter collaborative study covers almost half of the Dutch population. This, and the long inclusion period, resulted in a large patient sample size; this is a major strength in the context of the generalizability of our findings for the pediatric population. Our study also has several shortcomings. First are the retrospective study design and a significant amount of missing data. Second, there is possible overestimation of the SCA population by inclusion of unresolved etiologies without performing all necessary investigations to rule out non-cardiac etiologies. Further, inclusion started upon hospital admission. SCD at home and transport to a non-study hospital has not been considered, potentially underestimating the number of SCA. Also, as a consequence, the age distribution could have been biased because mainly infants have (unwitnessed) events in private homes, which are more often futile to resuscitative efforts, thus never reaching a hospital [3, 41].

## Conclusion

SCA etiology remained largely unresolved in infants and in one-third of children aged ≥ 1 year, but essential diagnostics were often not performed. Over one-fifth of SCA patients underwent prior cardiac evaluation, but this did not lead to recognition of a cardiac condition predisposing to SCA in all of them. The diagnostic post-SCA approach should be improved and the proposed standardized pediatric post-SCA diagnostics protocol (Fig. 3) may ensure a consistent and systematic evaluation process increasing the diagnostic yield and enhancing our understanding of SCA and our ability to prevent SCA in family members with ICC. Future studies are needed to improve the recognition of children at risk for SCA.

### Supplementary Information

Below is the link to the electronic supplementary material.

Supplementary file1 (DOCX 62.3 KB)

## Abbreviations

ARREST: AmsteRdam REsuscitation Studies
ECMO: Extracorporeal membrane oxygenation
ICC: Inherited cardiac condition
ICD: Implantable cardioverter-defibrillator
OHCA: Out-of-hospital cardiac arrest
ROC: Return of circulation
SCA: Sudden cardiac arrest
SCD: Sudden cardiac death
SUDI: Sudden Unexplained Death in Infancy
VF: Ventricular fibrillation
