# Supplementary material for: Sudden cardiac arrest in infants and children: proposal for a diagnostic workup to identify the etiology. An 18-year multicenter evaluation in the Netherlands
Source: Eur J Pediatr. 2023 Oct 27;183(1):335–44. doi: 10.1007/s00431-023-05301-9 (PMC10858117; doi:10.1007/s00431-023-05301-9)
Supplement: Supplementary file 1 — Supplementary file1 (DOCX 62.3 KB) [file 431_2023_5301_MOESM1_ESM.docx]

**Sudden cardiac arrest in infants and children: proposal for a diagnostic workup to identify the etiology.** *An 18-year multicenter evaluation in the Netherlands.*

Ashley M. Bakker MD¹*, Marijn Albrecht MD^2^*, Bas J. Verkaik MD^3^, Rogier C. J. de Jonge MD, PhD^2^, Corinne M. P. Buysse MD, PhD^2^, Nico A. Blom MD, PhD ^4,5^, Lukas A. J. Rammeloo MD^4,5^, Judith M. A. Verhagen MD, PhD^6^, Maaike A. Riedijk MD, PhD^7^, Sing C. Yap, MD, PhD^8^, Hanno L. Tan MD, PhD^3,9^, Janneke A. E. Kammeraad MD, PhD^1^

*^1^Department of Pediatric Cardiology, Erasmus MC Sophia Children's Hospital, Rotterdam, The Netherlands
^2^Department of Neonatal and Pediatric Intensive Care, Division of Pediatric Intensive Care, Erasmus MC Sophia Children's Hospital, Rotterdam, The Netherlands
^3^Heart Center, Department of Clinical and Experimental Cardiology, Amsterdam UMC, University of Amsterdam, Cardiovascular Sciences, Amsterdam, The Netherlands
^4^The Center for Congenital Heart Disease Amsterdam–Leiden, Amsterdam, The Netherlands
^5^Department of Pediatric Cardiology, Amsterdam University Medical Center, Location AMC, Amsterdam, The Netherlands
^6^Department of Clinical Genetics, Erasmus MC, University Medical Center Rotterdam, Rotterdam, The Netherlands
^7^Department of Pediatric Intensive Care, Emma Children's Hospital, Amsterdam University Medical Center, Amsterdam, The Netherlands
^8^Department of Cardiology, Erasmus MC, Rotterdam, The Netherlands
^9^Netherlands Heart Institute, Utrecht, The Netherlands
*AB and MA contributed equally*

Supplementary Table 1. Definitions adapted from the 2020 APHRS/HRS expert consensus statement on SCA (16).

| Term | Definition |
| --- | --- |
| Out-of-hospital cardiac arrest (OHCA) | Loss of functional cardiac mechanical activity in association with an absence of systemic circulation, occurring outside of a hospital setting |
| Return of Circulation (ROC) | Sustained ROC is deemed to have occurred when chest compressions are not required for 20 consecutive minutes and signs of circulation persist either with spontaneous circulation (ROSC) or supported by extracorporeal membrane oxygenation (ECPR). |
| Sudden cardiac arrest (SCA) | Sudden cessation of cardiac activity with hemodynamic collapse, typically due to sustained ventricular arrhythmia |
| Sudden cardiac death (SCD) | Death that occurs within 1 hour of onset of symptoms in witnessed cases, and within 24 hours of last being seen alive when it is unwitnessed |
| Sudden Unexplained Death in Infancy (SUDI) | Unexplained sudden death occurring in an individual younger than 1 year with negative pathological and toxicological assessment. |

Supplementary Table 2. Medical history, prior cardiac evaluation & family history in relation to etiology of SCA.

|  | Overall | | | Primary arrhythmogenic disorders | | | Cardiomyopathy | | | Congenital heart anomaly | | | Other cardiac causes | | | Unresolved | | |
| --- | --- | --- | --- | --- | --- | --- | --- | --- | --- | --- | --- | --- | --- | --- | --- | --- | --- | --- |
|  | (n = 172) | | | (n = 37) | | | (n = 28) | | | (n = 9) | | | (n = 15) | | | (n = 83) | | |
| **Prior to SCA** | n^a^ |  |  | n^a^ |  |  | n^a^ |  |  | n^a^ |  |  | n^a^ |  |  | n^a^ |  |  |
| **Evaluation by a pediatric cardiologist^b^** | 172 | 38 | 22% | 37 | 12 | 32% | 28 | 11 | 39% | 9 | 2 | 22% | 15 | 2 | 13% | 83 | 11 | 13% |
| **A) *No pre-SCA cardiac diagnosis^b^*** | 38 | 4 | 11% | 37 | 0 | 0% | 28 | 1 | 4% | 9 | 0 | 0% | 15 | 0 | 0% | 83 | 3 | 4% |
| ***B)*** ***Pre-SCA cardiac diagnosis or major event^b^*** | 38 | 34 | 89% | 37 | 12 | 32% | 28 | 10 | 36% | 9 | 2 | 22% | 15 | 2 | 13% | 83 | 8 | 10% |
| *Primary arrhythmogenic disorders*^b^ | 34 | 6 |  | 12 | 5 |  | 10 | 0 |  | 2 | 0 |  | 2 | 0 |  | 8 | 1 |  |
| *Cardiomyopathy*^b^ | 34 | 10 |  | 12 | 0 |  | 10 | 9 |  | 2 | 0 |  | 2 | 1 |  | 8 | 0 |  |
| *Congenital heart anomaly*^b^ | 34 | 19 |  | 12 | 6 |  | 10 | 2 |  | 2 | 2 |  | 2 | 1 |  | 8 | 8 |  |
| *Other*^b^ | 34 | 4 |  | 12 | 2 |  | 10 | 1 |  | 2 | 1 |  | 2 | 0 |  | 8 | 0 |  |
| *Cardiac intervention*^b^ | 34 | 6 |  | 12 | 2 |  | 10 | 2 |  | 2 | 0 |  | 2 | 0 |  | 8 | 2 |  |
| *Sudden cardiac arrest*^b^ | 34 | 3 |  | 12 | 2 |  | 10 | 0 |  | 2 | 0 |  | 2 | 0 |  | 8 | 1 |  |
|  |  |  |  |  |  |  |  |  |  |  |  |  |  |  |  |  |  |  |
| **Neurologic medical history^b^** | 170 | 25 | 15% | 37 | 6 | 16% | 28 | 4 | 14% | 9 | 0 | 0% | 15 | 1 | 7% | 81 | 14 | 17% |
| *Epilepsy*^b^ | 25 | 12 |  | 6 | 2 |  | 4 | 1 |  | 0 | 0 |  | 1 | 1 |  | 14 | 8 |  |
| *Psychomotor retardation*^b^ | 25 | 8 |  | 6 | 3 |  | 4 | 2 |  | 0 | 0 |  | 1 | 0 |  | 14 | 3 |  |
| *Other*^b^ | 25 | 5 |  | 6 | 1 |  | 4 | 1 |  | 0 | 0 |  | 1 | 0 |  | 14 | 3 |  |
|  |  |  |  |  |  |  |  |  |  |  |  |  |  |  |  |  |  |  |
| **Positive family history^b^** | 118 | 15 | 13% | 30 | 5 | 17% | 27 | 9 | 33% | 6 | 0 | 0% | 13 | 0 | 0% | 42 | 1 | 2% |
| *Sudden death <40 years*^b^ | 15 | 5 |  | 5 | 2 |  | 9 | 3 |  | 0 | 0 |  | 0 | 0 |  | 1 | 0 |  |
| *Primary arrhythmogenic disorders*^b^ | 15 | 7 |  | 5 | 3 |  | 9 | 3 |  | 0 | 0 |  | 0 | 0 |  | 1 | 1 |  |
| *Non-ischemic cardiomyopathy*^b^ | 15 | 6 |  | 5 | 0 |  | 9 | 6 |  | 0 | 0 |  | 0 | 0 |  | 1 | 0 |  |

*A child could have had multiple pre-existing conditions so the total number per category is not necessarily the sum of the subheadings.
Abbreviations: SCA = Sudden cardiac arrest
^a^ Number of subjects in whom the variable was obtained.
^b^ Number of subjects (%)*

*Supplementary Table 3. Proposed post-SCA diagnostic protocol in paediatric SCA, in addition to Figure 3.*

**PATIENT HISTORY**

1. Medical (cardiac) history + medication use

- Cardiac history:
  □ None
  □ Arrhythmia, namely...……………………………………………………………………………………………………………………………….
  □ Cardiomyopathy, namely...………………………………………………………………………………………………………………………..
  □ Congenital structural heart defect, namely……………………………………………………………………………………………….
  □ Cardiac surgery, namely...………………………………………………………………………………………………………………………….
  □ Previous evaluation by pediatrician (cardiologist) due to……………………………………….....................................
  □ Other, namely...…………………………………………………………………………………………………………………………………………
- Other medical history:
  □ None
  □ Epilepsy (if present: confirmed by EEG yes/no)
  □ (Exercise-induced) asthma
  □ Hyper/hypothyroidism
  □ Developmental disorders/behavioural problems
  □ Other, namely...…………………………………………………………………………………………………………………………………………
- Medication
  □ Yes, namely...…………………………………………………………………………………………………………………………………………….
  □ No

**EVENT HISTORY**

2. Time of event………………………………………………………………………………………………………………………………………………….

1. Witnesses
   □ Yes (witnessed OHCA <1 hour after onset of symptoms). By whom:………………………………………………………….
   □ No (unwitnessed, but seen alive <24 hours without prior symptoms)
2. Location……………………………………………………………………………………………………………………………………………………….
3. Circumstances
   □ At rest (during sleep)
   □ Activities of daily living (awake, not in bed, non-active play)
   □ (Post) moderate to active physical exertion
   □ Swimming/diving
   □ Stress
   □ Intense emotion
   □ Fear/startle
   □ Fever episode
   □ Auditory stimulus
   □ Unknown
   □ Other, namely...…………………………………………………………………………………………………………………………………………
4. Intoxication
   □ Alcohol
   □ Drugs
   □ Caffeine
   □ Smoking
   □ Any medication present at home (specify + dosage)…………………………………………………………………………………
5. Prodromal symptoms (<24 hours before OHCA)

- Cardiac symptoms
  □ (Pre) syncope/seizure
  □ Palpitations
  □ Chest pain
  □ Dizziness
  □ Shortness of breath
  □ Reduced exercise tolerance (different from other children or difficult to drink in children <1 year (e.g., sweating)
- Non-specific symptoms:
  □ General malaise
  □ Fever
  □ Gastrointestinal symptoms, namely………………………………………………………………………………………………………….
  □ Respiratory symptoms, namely…………………………………………………………………………………………………………………

1. Antecedent symptoms (>24 hours before Sudden Cardiac Arrest)

- Cardiac symptoms (explained yes/no, under what circumstances (see question 5)
  □ (Pre) syncope/seizure
  □ Palpitations
  □ Chest pain
  □ Dizziness
  □ Shortness of breath
  □ Reduced exercise tolerance (different from other children or difficult to drink in children <1 year (e.g., sweating)
- Non-specific symptoms:
  □ General malaise
  □ Fever
  □ Gastrointestinal symptoms, namely………………………………………………………………………………………………………….
  □ Respiratory symptoms, namely…………………………………………………………………………………………………………………

1. Family History
   □ Sudden unexplained death before the age of 50
   □ Arrhythmias (explicit pacemaker/ICD: yes/no)
   □ Cardiomyopathy
   □ Heart failure
   □ Unexplained syncope/seizure
   □ Epilepsy (confirmed by EEG yes/no)
   □ Congenital bilateral deafness

NB: For a positive family history, inquire in detail: (e.g.: familial relationship to the patient; age of onset) ……………………………………………………………………………………………………………………………………………………………………. …………………………………………………………………………………………………………………………………………………………………….…………………………………………………………………………………………………………………………………………………………………….……………………………………………………………………………………………………………………………………………………………………. .……………………………………………………………………………………………………………………………………………………………………
.……………………………………………………………………………………………………………………………………………………………………
.……………………………………………………………………………………………………………………………………………………………………
.……………………………………………………………………………………………………………………………………………………………………
.……………………………………………………………………………………………………………………………………………………………………
.……………………………………………………………………………………………………………………………………………………………………
.……………………………………………………………………………………………………………………………………………………………………

**CLINICAL DIAGNOSTICS**

☐ Save AED and ECG monitoring registrations around the OHCA event

☐ Blood culture (Note: take blood sample before potential initiation of ECMO!)
 □ Anaerobic culture bottle
 □ Aerobic culture bottle
 Specify in the request: prolonged culturing (to also culture slow growers/fungi)

☐ Toxicological investigation (Note: take blood sample before potential initiation of ECMO!)
 □ blood
 □ urine (for substances like GHB etc. These cannot be detected in blood)

☐ Blood chemistry (if circulation is present or <2 hours after death)
 □ Electrolytes, kidney function, liver function, cortisol, osmolality, CK, CRP, glucose, NT pro-BNP
 □ Complete blood count
 □ Blood gas, lactate (capillary or arterial)

☐ (Future) DNA investigation (Note: take blood sample before potential initiation of ECMO).
 □ DNA storage, do not initiate tests yet.
 □ (Alternative: skin biopsy (in culture medium, at room temperature; not in the refrigerator!)

☐ Metabolic investigation (Collect as soon as possible)
 □ blood
 □ urine

☐ Optional spare material

☐ Exclude non-cardiac causes based on patients history/physical examination/ additional examination:
o Trauma, infection, intoxication, electrolyte abnormalities
o Neurologic disorders (consider CT of the brain)
o Metabolic disorder

*If circulation returns*:

In consultation with pediatric cardiologist:
☐ 12-lead resting ECG (repeat several times during hospital admission)
☐ Echocardiogram (cardiac anatomy / ventricular function / myocardium / coronaries / pulmonary hypertension)
☐ Continuous Rhythm monitoring
☐ Cardiac exercise stress testing

*Consider*: ADDITIONAL DIAGNOSTICS
☐ Drug provocation tests (Ajmaline test, Epinephrine test) and fever ECG
☐ Signal-averaged ECG
☐ Cardiac CT angiography (coronaries)
☐ Cardiac MRI with late enhancement, preferably a couple of days after resuscitation (myocardial fibrosis, ventricular function, dimensions and wall motion, fatty infiltration, signs of myocarditis, aneurysms)
☐ Electrophysiological study
☐ DNA investigation. Determine which DNA investigation during multidisciplinary team meeting discussing the phenotype

☐ Autopsy if deceased during hospital admission (including macro-/microscopic pathology of the heart)

*If circulation does not return*:

☐ Autopsy (including macro-/microscopic cardiac autopsy)
 o If no permission for autopsy: consider total body MRI

☐ If natural death is not confirmed and forensic autopsy is indicated, consult the forensic physician to request a forensic autopsy including a cardiac autopsy.

☐ Consider consultation of (pediatric) cardiology

☐ Consider cardiac evaluation of 1st degree family members

☐ Consider DNA investigation. Determine which DNA investigation during multidisciplinary team meeting discussing the phenotype
